# Regulatory Changes of N-Acetylgalactosamine Terminal Sugar in Early Mouse Embryonic Paraxial Mesenchyme

**Published:** 2012-08-31

**Authors:** Mohammad Mehdi Hassanzadeh Taheri, Ali Reza Ebrahimzadeh Bideskan, Mohammad Reza Miri

**Affiliations:** 1. Department of Anatomy, School of Medicine, Birjand University of Medical Sciences, Birjand, Iran; 2. Department of Anatomy, School of Medicine, Mashad University of Medical Sciences Mashad, Iran; 3. School of Health, Birjand University of Medical Sciences, Birjand, Iran

**Keywords:** Embryonic, Glycoconjugate, Terminal Sugar, Mesenchyme

## Abstract

**Objective:**

The development of vertebrae is a complex phenomenon that is correlated with distinct morphological and biochemical alterations in the paraxial mesenchyme and glycoconjugates. The purpose of this study is to investigate the glycosylation pattern in paraxial mesenchyme-forming vertebrae by using the lectin histochemical technique.

**Materials and Methods:**

In this descriptive-analytic study, B4G fixed paraffin sections of 9 to 15 day Balb/c mouse embryos were processed for histochemical studies using seven different HRP-labelled lectins: Glycin max (SBA), Maclura pomifera (MPA), Wistaria floribunda (WFA), Vicia villosa (VVA) which all of them are specific for N-acetylgalactosamine (GalNAc), Ulex europius (UEA1, binds to α-L-fucose), wheat germ agglutinin (WGA, binds to sialic acid), and Griffonia simplicifolia (GSA1-B4, binds to galactose terminal sugars). The sections were observed separately by three examiners who were blinded to the lectins. Grading was done according to the intensity of the tested lectins’ reactions with the specimen, from negative (-) to severe (+++). Data was analysed with SPSS software (version 11.5) and the non-parametric Kruskal Wallis test; p<0.05 was considered significant.

**Results:**

Our findings showed that among the tested lectins, only GalNAc residue sensitive lectins showed regulated changes in paraxial mesenchyme. Reactions of WFA and MPA lectins with paraxial mesenchyme were severe on GD9. Reactions of WFA continued to GD15 constantly, while MPA reactions continued strongly to GD12, significantly decreased thereafter (p<0.001), and then disappeared. VVA and SBA bindings initiated weakly on GD10 and continued to GD12 without changing. These reactions increased significantly (p<0.001) thereafter, became severe to GD14, and later disappeared. The other tested lectins did not reveal regulated changes.

**Conclusion:**

According to these findings it can be concluded that only the GalNAc terminal sugar showed temporally regulated changes during the early embryonic development of vertebrae in mice. Therefore it most likely plays a key role (s) in the development of vertebrae, especially in the conversion of mesenchymal cells into chondroblasts. The other tested terminal sugars may have no role in this phenomenon.

## Introduction

During early development of the vertebrate embryo, the primary segmented tissue of the body axis is the paraxial mesoderm that lies bilaterally to the axial organs, neural tube, and notochord (1-3). The term for the segmented aggregates cell is somites (2, 3). Next these cells differentiate and the ventro-medial portion changes into its mesenchymal form (sclerotome), which are the precursor cells for vertebrae, ribs, and intervertebral discs (2-5).

The molecular control of somite differentiation and sclerotome proliferation and differentiation has only somewhat been eluciated in both chicken and mouse models (6). The sonic hedgehog (shh) signaling molecule (4, 7) and several transcription factors including members of the *pax* and *sox* families, are required for these phenomena to occur (8-10). It is well known that the notochord induces sclerotomal cells by secretion of inductive factors such as Shh and Noggin, among others, to migrate, proliferate, differentiate, and adhere to one another for creating vertebrae (7, 8, 11, 12). The development of the vertebral column in vertebrates is a complex phenomenon that is correlated with distinct morphological and biochemical alterations of the mesenchymal component, such as: induction, cell migration, differentiation, recognition, adhesion, aggregation, condensation, and transformation which is regulated by different subsets of morphogenes (6, 7, 13). It can be assumed that structural modification of glycoconjugates are involved in these processes (13).

Glycoconjugates are present at animal cell surfaces and in the extracellular matrix (ECM) (14). Terminal sugars of these macromolecules correlate with essential functions of cellular interactions such as cell recognition, receptor function, cell adhesion, and migration (15). They can be detected histochemically using natural polypeptides (lectins), which are obtained from plant or animal sources (16).

Despite extensive studies on the use of lectin histochemistry in the development of certain organs [small and large intestine (17), testis (18), prostate (19), placenta (20), and differentiation of chondrocytes (21, 22), few studies have focused on the development of the vertebral column by this technique.

Studies focusing on the expression of glycoconjugates and their role (s) in vertebral column development are limited. In a study conducted by Götz and colleagues in 1991, the lectin binding pattern in the embryonal and early fetal human vertebral column was studied (23). In 1993 they reported on the lectin binding pattern in the human paraxial mesenchyme (24), and in 2001 they studied the distribution of glycoconjugates in the notochord and axial mesenchyme of human embryos (13). Bagnall and Sanders (1989) studied the binding pattern of PNA lectin associated with sclerotome migration during the formation of vertebral axis in chick embryos (25). Quondamatteo et al. noted that in undulated mouse mutants (substitution in the *Pax1* gene), a malformation not only occurred in the vertebral column, but the glycosylation pattern was also altered in normal and malformed organs (26). Moiin et al. studied changes in the terminal sugars of glycoconjugates in precursor cartilage that formed the vertebrae in rat embryos (27). Expression and changes of some terminal carbohydrate residues of glycoconjugates were studied during the conversion of mesenchyme to cartilage forming vertebrae in mouse embryos via WFA and OFA lectins by Nikravesh et al. in 2002 (28).

The aim of the present study was to investigate the lectin binding pattern of paraxial mesenchyme forming vertebrae and their possible alterations during early morphogenesis in Balb/c mouse embryos by means of the lectin histochemistry method.

## Materials and Methods

### Preparation of mouse embryos

For this descriptive-analytic study, 30 sexually mature adult virgin female Balb/C mice average age 7-8 weeks age that weighed 25-30 g and 15 fertile males of the same strain were obtained from the Razi Institute in Mashhad. They were individually caged in an animal house for two weeks before examination for adaptation at the Mashhad University of Medical Sciences, Mashhad, Iran.

Animals were fed standard laboratory chow and water ad libitum, under controlled conditions (12 hour light/dark cycle, 24 ± 2℃, 50-55% relative humidity).

Animals were mated overnight (2 female + 1 male) in separate cages and subsequently examined the next morning for the presence of a vaginal plug that confirmed pregnancy; the same day was regarded as gestational day 0 (GD0). From GD9 to GD15, four pregnant mice were randomly sacrificed by cervical dislocation for each stage. Embryos were dissected and freed carefully from the uterus and extraembryonic membranes (mean numbers of embryos were 8 for each pregnant mouse). All protocols for the animal experiments were approved by the Institution’s Animal Care Committee of the Mashhad University of Medical Sciences.

### Preparation of tissue sections

Whole embryos were washed in normal saline and subsequently fixed in B4G fixative (1% sodium acetate, 6% mercuric chloride, 0.1% glutaraldehyde) overnight at room temperature. After specimen dehydration by passing through a series of successive graded ethanols, the embryos were cleared with xylene and embedded in paraffin blocks. A rotary microtome were used to cut transverse serial sections of 6 mm thicknesses.

### Lectin histochemistry

Seven horseradish peroxidase-conjugated lectins (Table 1) were purchased from Sigma Chemical Company (USA) and diluted with phosphate buffered saline (PBS) to 10-20 µg of lectin in 0.1 M PBS (29-31). Lectins consisted of: Glycin max (SBA), Maclura pomifera (MPA), Wistaria floribunda (WFA) and Vicia villosa (VVA) which all of them are specific for N-acetylgalactosamine (GalNAc), Ulex europius (UEA1, binds to α-L-fucose), wheat germ agglutinin (WGA, binds to sialic acid) and Griffonia simplicifolia (GSA1-B4, binds to galactose terminal sugars).

Five sections of the thoracic region of each embryo were chosen randomly. The specimens were deparaffinized in xylene and hydrated by passing in a series of reduced grades ethanol. They were then treated with Lugol’s solution to remove mercuric salts prior to histochemical staining. Thereafter the sections were rinsed for 10 minutes in 0.1 M PBS (pH=7.4). Intrinsic peroxidase activity was blocked with hydrogen peroxide-methanol (1: 100), for 45 minutes in the dark (13, 30). All sections were incubated with chosen lectins in a humid chamber at room temperature for 2 hours. After extensive rinising in PBS, sections were incubated in diaminobenzidine (DAB)-hydrogen peroxidase substrate medium (pH=7.0), for 15 minutes at room temperature. All sections were counterstained with a 1% solution of alcian blue at pH=2.5 for 5 minutes (29-31). Finally the sections were dehydrated, cleared, and mounted on glass slides. Controls for lectin staining were carried out as such: substitution of unconjugated lectin for lectin-HRP conjugates and exposure of sections to HRP and substrate medium without lectin.

The affinities of the tested lectins were separately observed by three examiners blinded to the lectins under a light microscope (Olympus AH-2), and grading was done according to the intensity of lectin reactions with the specimen, from negative (-) to severe (+++) (29-31). The medium of the suggested grades for each lectin on each day was calculated.

Comparisons were then made among the various tested lectins and also across the different types of tissues and stages of development. Data were analysed by using SPSS software (version 11.5) and non-parametric Kruskal Wallis test; differences less than p<0.05 were considered significant.

**Table 1:** List of lectins that were tested in the present study and their main sugar specificities


Lectin tested	Abbreviation	Carbohydrate-binding specificity

Hairy winter vetch (Vicia villosa agglutinin)	VVA	β Gal 1→ 3 α GalNac>Gal
Glycine max (Soybean agglutinin)	SBA	α/ β- D-GalNac> α/ β-D-Gal
Wistaria floribunda	WFA	α -D- GalNac & β -D-GalNac
Maclura pomifera agglutinin	MPA	Gal- β (1→ 3) -GalNac
Ulex europeus agglutinin	UEA-1	α- L- Fuc (α 1→ 2) Gal (β 1→4) GlcNac β
Triticum vulgaris (Wheat germ agglutinin)	WGA	[(β1→ 4) -D-GlcNac] 2 or 3 Sialic acid
Griffonia simplicifolia	GSA1-B4	D-Gal


Fuc; Fucose, Gal; Galactose, GalNac; N-Acetylgalactosamine and Glc; Glucose.

## Results

Somatic cell differentiation was noted in the craniocaudal direction with time progression, their histomorphological and histochemical changes were evaluated by comparing similar sections in different GDs.

### Histomorphology

**Fig 1:**
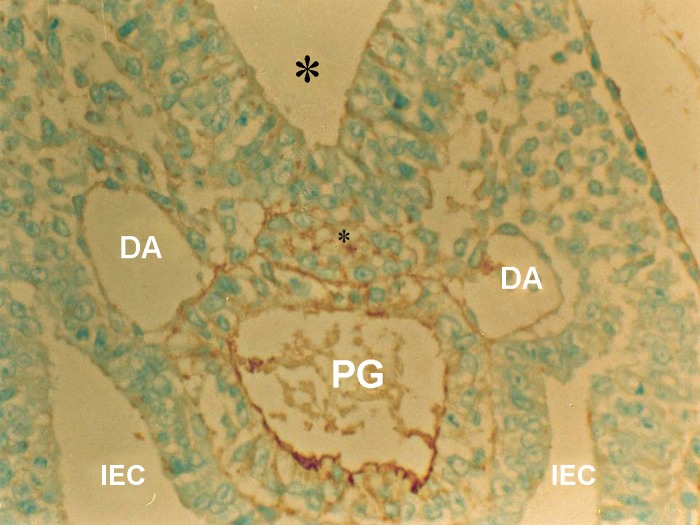
Photomicrograph of GD9 mouse embryo, cross-section of paraxial mesenchyme, notochord, and ventral portion of neural groove incubated with VVA lectin. Notochordal process (Small star), Neural groove (Big star), DA; Dorsal aorta, PG; Primitive gut, IEC; Intera embryonic coelum Intra-embryonic coelom (×400).

**Fig 2:**
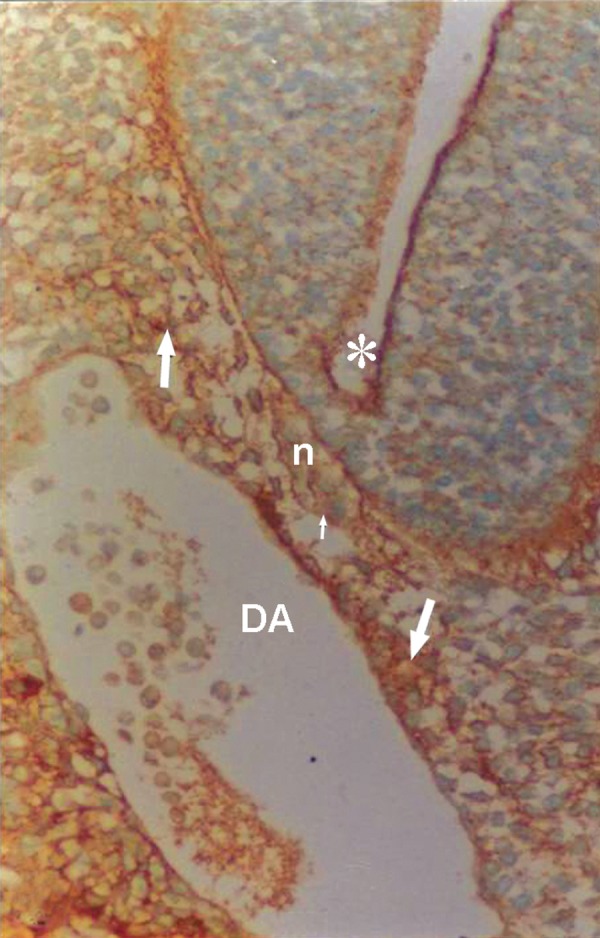
Photomicrograph of GD10 mouse embryo, cross-section of paraxial mesenchyme, notochord, and ventral portion of neural tube incubated with WFA lectin. Notochordal plate (n), its sheath (small arrow), neural tube (star), DA; Dorsal aorta. Severe reaction observed in paraxial mesenchyme (arrow; ×200).

**Fig 3:**
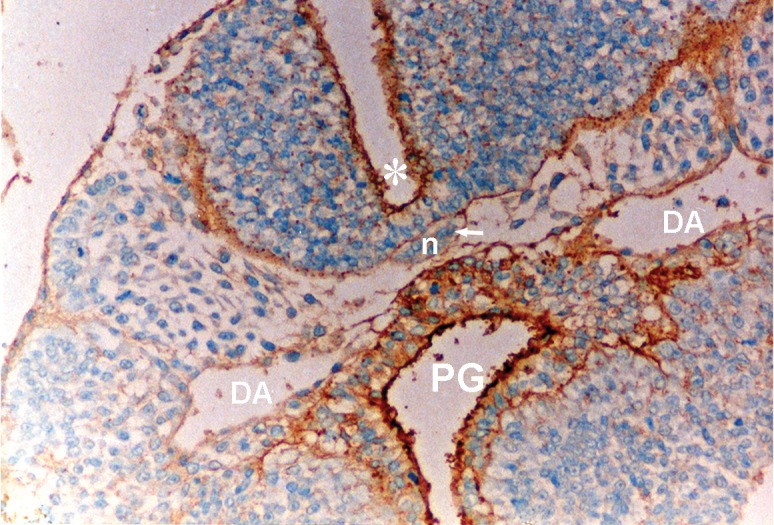
Photomicrograph of GD10 mouse embryo, cross-section of paraxial mesenchyme, notochord, and ventral portion of neural tube and primitive gut, incubated with SBA lectin. Notochordal plate (n), its sheath (arrow), neural tube (star), DA; Dorsal aorta, PG; Primitive gut (×200) .

**Fig 4:**
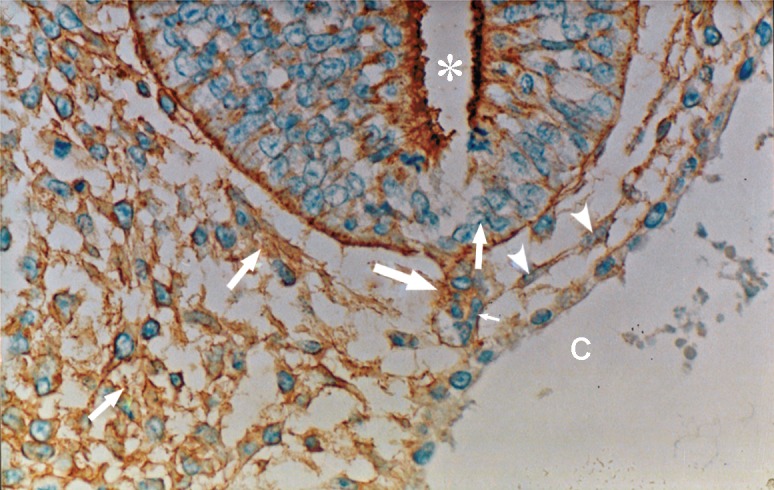
Photomicrograph of GD10 mouse embryo, cross-section of paraxial mesenchyme, notochord, and ventral portion of neural tube, incubated with MPA lectin. Notochord (big arrow), its sheath (small arrow), neural tube (star), C; Coelum Coelom. Severe reaction observed in mesenchymal surfaces (head arrow) and in ECM (medium arrow) (×200).

**Fig 5:**
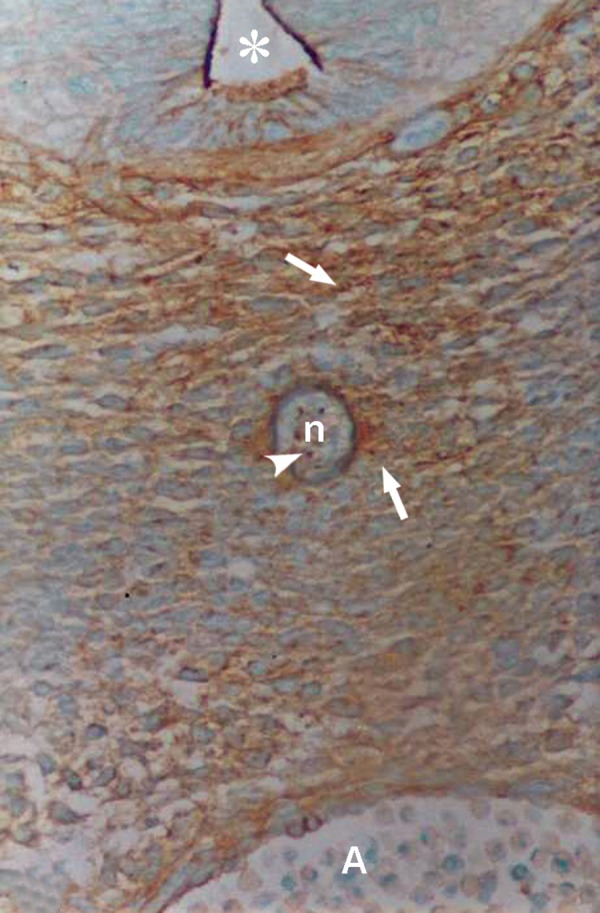
Photomicrograph of GD12 mouse embryo, cross-section of paraxial mesenchyme, notochord, and ventral portion of neural tube incubated with WFA lectin. Notochord (n), reaction of its cells (head of arrow), neural tube (star), A; Aorta. Severe reaction observed in paraxial mesenchyme (arrow) (×200).

**Fig 6:**
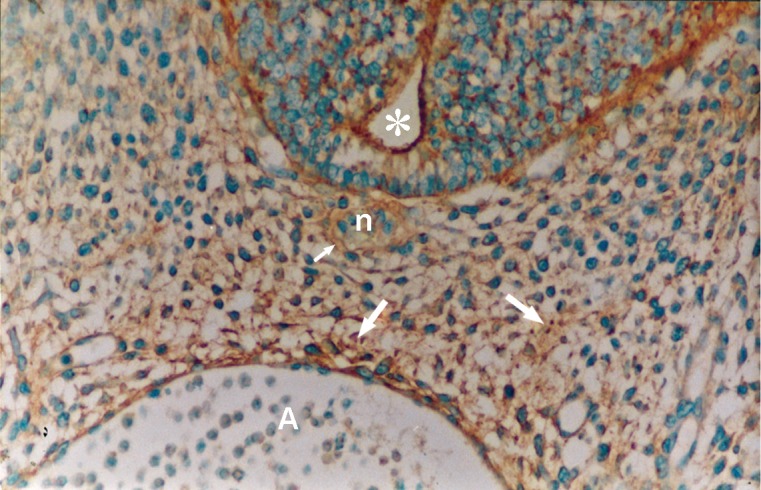
Photomicrograph of GD11 mouse embryo, cross-section of paraxial mesenchyme, notochord, and ventral portion of neural tube, incubated with MPA lectin. Notochord (n), its sheath (small arrow), neural tube (star), A; Aorta. Severe reaction observed in paraxial mesenchyme (big arrow) (×200).

**Fig 7:**
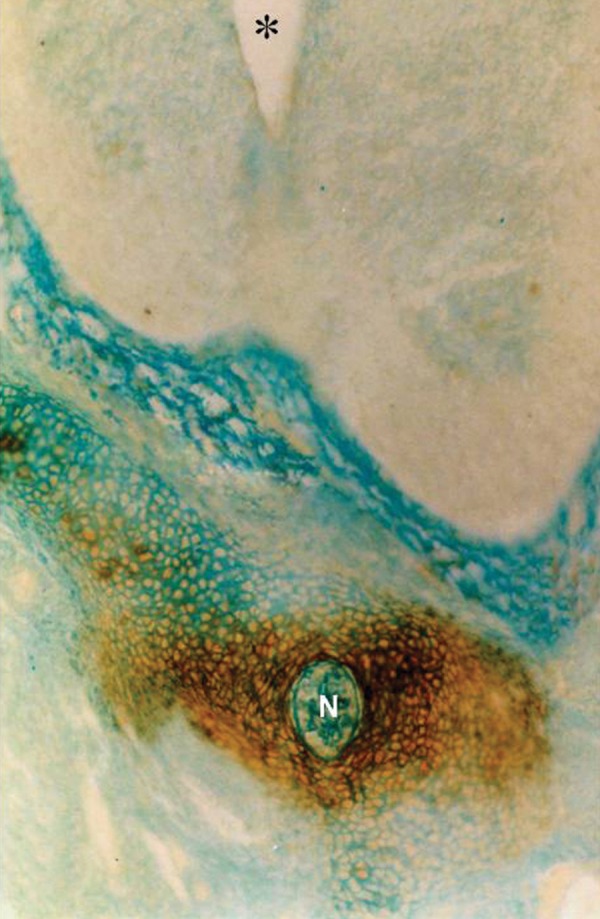
Photomicrograph of GD13 mouse embryo, cross-section of paraxial mesenchyme, notochord, and ventral portion of neural tube, incubated with VVA lectin. Severe reaction observed in paraxial mesenchyme around the notochord. Notochord (N), Neural tube (star) (×200).

**Fig 8:**
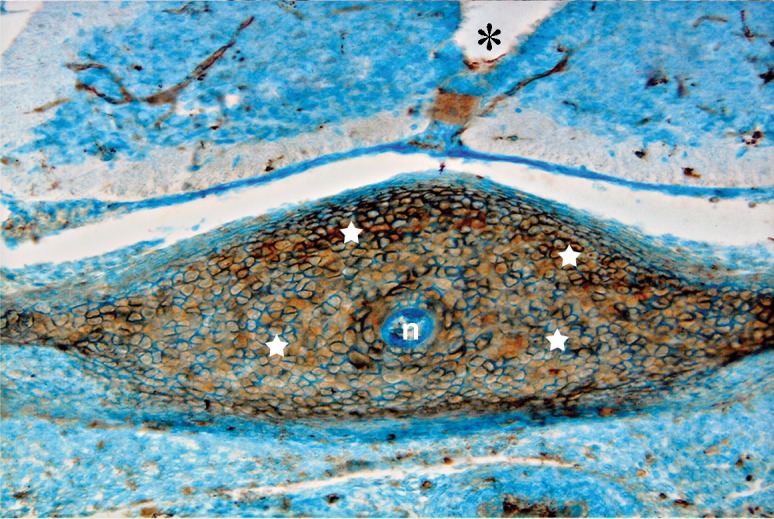
Photomicrograph of GD13 mouse embryo, cross-section of paraxial mesenchyme, notochord, and ventral portion of neural tube, incubated with SBA lectin. Notochord (n), neural tube (star). Severe reaction observed in paraxial mesenchyme (white stars) (×200).

**Fig 9:**
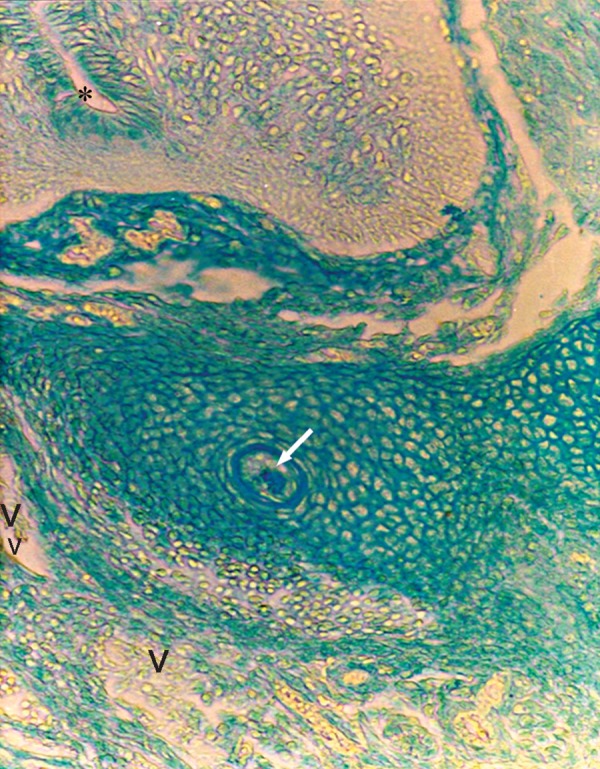
Photomicrograph of GD13 mouse embryo, cross-section of paraxial mesenchyme, notochord, and ventral portion of neural tube incubated with GSA1-B4 lectin. Notochord (arrow), neural tube (star), V; Vessel (×200).

Using conventional staining (H&E), the charactristic pattern of somatic cell differentiation and development of vertebrae occurred as follows:

1. On GD9 the epithelial cells began to detach from the ventro-medial portion of the somite and transformed into mesenchymal cells, termed sclerotom (Fig 1).
2. The sclerotomal cells initiated migration to the periphere of the axial structures, notochord, and neural tube, and accumulated around them (Figs 2-4).
3. These mesenchymal cells were condensed, and gradually a prechondroginous blastema formed without a clearly defined margin (Figs 5, 6).
4. After 2-3 days, the embryonic cartilage was composed of closely packed chondrocytes and ECM (Figs 7-9).

### Lectin histochemistry

Reactions of glycoconjugates with tested lectins on different GDs were studied and findings have been summarized in table 2.

### WFA

A severe reaction of paraxial mesenchyme was found in all specimens investigated (Figs 2, 5). At high magnification this staining was present on the cytoplasm, cell surface, and ECM. The notochord, its sheath, vessel wall, and neuroepithelial cells (especially at the apical plasma membrane of the luminal cells) also showed strong staining.

### MPA

Reactions of this lectin were similar to those of WFA, but continued only to GD12 and thereafter decreased significantly at GD13 (p<0.001) and disappeared (Figs 4, 6).

### SBA

This lectin reacted weakly with paraxial mesenchyme on GD10 and continued without change to GD12, after which it increased significantly (p<0.001) and was assessed as strong. This reaction decreased significantly again on GD15 (p<0.001) and became negative. Reactions of the notochord and its sheath were moderate, while neuroepithelial cells, especially at the apical plasma membrane of the luminal cells showed a strong reaction (Figs 3, 8).

### VVA

**Fig 10:**
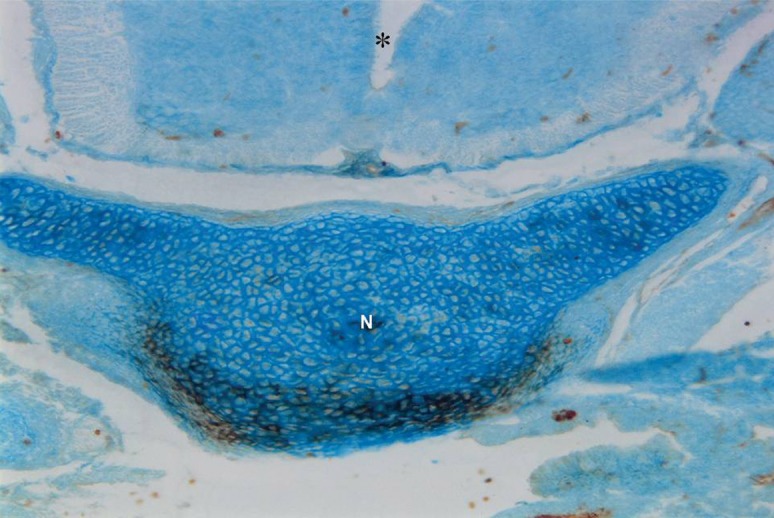
Photomicrograph of GD15 mouse embryo, cross section of paraxial mesenchyme, notochord and ventral portion of neural tube incubated with VVA lectin. Notochord (N), Neural tube (star), Reaction of this lectin restricted to the peripher zone of future vertebral body. Magnification =×200.

Reactions of this lectin were negative on GD9 (Fig 1), with a weak reaction on GD10 and continued without change to GD12. On GD13 these reactions changed significantly (p<0.001) and became strong, especially around the notochord, while the notochord reaction was negative on this day (GD 13) (Fig 7). This reaction continued to GD14 and changed significantly thereafter on GD15 (p<0.001) and then disappeared, except in the periphere of the future vertebral body (Fig 10). Reactions of the neural tube, notochord, and its sheath were negative on the GDs studied.

**Table 2:** Severity of reactions of tested lectins with paraxial mesenchyme on different Gestational dayss


Tested	GDs						

Lectins	9	10	11	12	13	14	15
VVA	-	+	+	+	+++	+++	-
SBA	-	+	+	+	+++	+++	-
WFA	+++	+++	+++	+++	+++	+++	+++
MPA	+++	+++	+++	+++	-	-	-
UEA1	-	-	-	-	-	-	-
WGA	-	+	+	+	+	+	+
GSA1-B4	-	-	-	-	-	-	-


Staining intensity based on estimated scale from 0 to +++, with: negative reaction; (-), weak; (+), moderate; (++) and severe; (+++).

### UEA1

**Fig 11:**
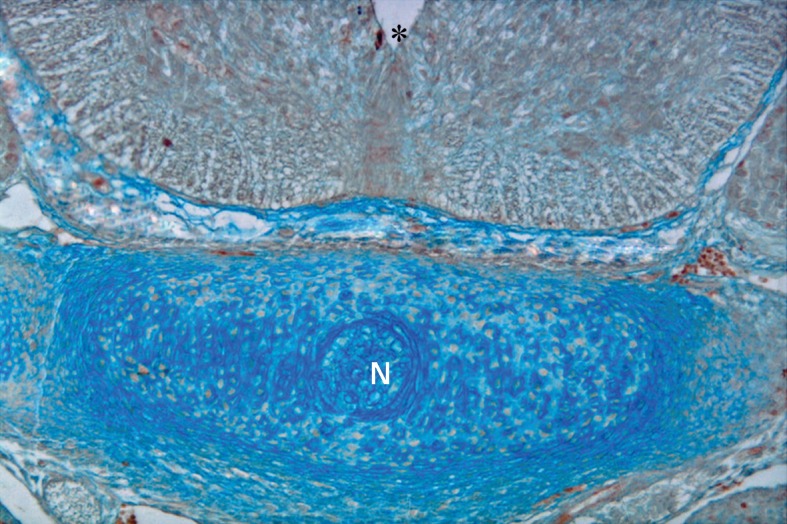
Photomicrograph of GD15 mouse embryo, cross-section of paraxial mesenchyme, notochord, and ventral portion of neural tube incubated with UEA1 lectin. Notochord (N), neural tube (star) (×200).

No reaction of this lectin was observed in the paraxial mesenchyme (Fig 11).

### WGA

**Fig 12:**
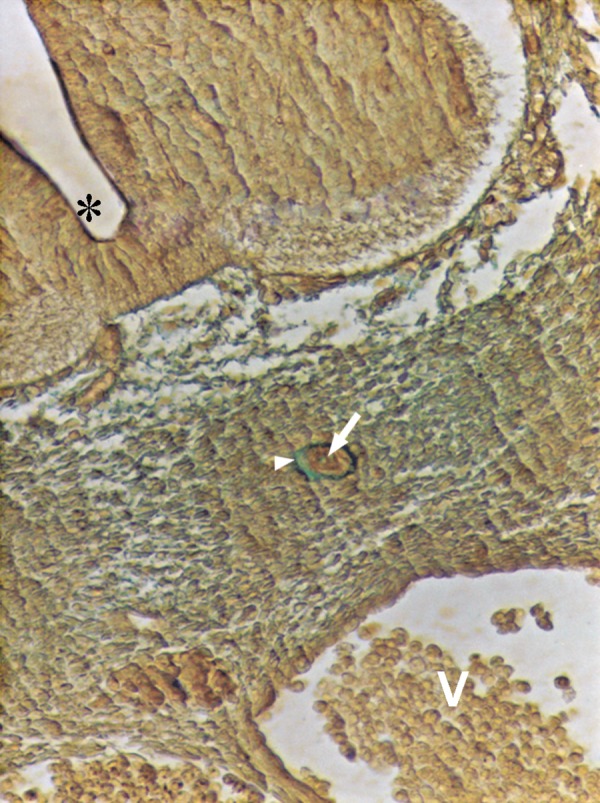
Photomicrograph of GD11 mouse embryo, cross-section of paraxial mesenchyme, notochord, and ventral portion of neural tube incubated with WGA lectin. Notochord (arrow), its sheath (head of arrow), neural tube (star), V; Vessel. Severe reaction only in the notochord (×200).

A weak, uniform staining was obseved in the paraxial mesenchymal cells during all GDs investigated (Fig 12).

### GSA1-B4

Binding of this lectin with paraxial mesenchyme was negative in the time period investigated (Fig 9).

## Discussion

Lectins serve as useful histochemical probes for studying the role of surface carbohydrate moieties in glycoconjugates during normal development, including the vertebral column. In this study seven different lectins were used to determine whether differences could be detected among various cell surfaces and ECM in the paraxial mesenchyme during normal development of the vertebral column in mouse embryos between GD9 and GD15.

In a previous study we found PNA positive terminal sugars in paraxial mesenchyme that changed regulatory and discussed (30); in the present study another subset of lectins were tested. Among the tested lectins, reactions of MPA and WFA appeared severe on GD9, reactions of WFA continued to GD15 without changing, while reactions of MPA continued to GD12 and disappeared later. Positive reactions of the paraxial mesenchyme with WFA lectin in mouse (28) and MPA in rat (27) and the contribution of their recognition of terminal sugars in the development of the vertebral column have been previously reported.

MPA and WFA lectins recognize GalNAc terminal sugar in spatial patterns, Gal-β (1→3)-GalNAc and α-or β-D-GalNAc respectively.

Zschäbitz et al. have shown that the expression of Gal-β (1→3)-GalNAc moieties during the development of the vertebrae in chondrocyte and perichodrium mesenchyme in rats (32), which are similar to our findings. MPA binding sites probably detect glycotopes on the laminin glycoprotein (33).

Reactions of VVA and SBA lectins which bind GalNAc terminal sugar in spatial patterns, β Gal (1→3) GalNAc and α/β-D-GalNAc respectively.

began in a similar manner they were weak on GD10 and did not change until GD12. Thereafter, these reactions increased significantly (p<0.001), appeared severely until GD14, and later disappeared (p<0.001). Positive moderate reactions of human paraxial mesenchyme with SBA lectin have been reported previously by Götz and Quondumatteo which confirm our findings (13). VVA lectin has been described as α-GalNAc-specific and has a strong preference for the α-GalNAc-Ser/Thr structure. Such sugar binding specificity would be extremely useful in detecting the initiation of 0-glycan biosynthesis (34).

UEA1 lectin was unreactive on the paraxial mesenchyme during the time period described. This finding has indicated that the terminant α-L-fuc is either completely lacking or masked by other sugars, such as neuraminic acid and thus is inaccessible to the lectin binding. α-L-fuc is a typical carbohydrate residue of the O-linked glycoprotein (34). In accordance with our results, Zschäbitz et al. did not find evidence for the presence of ‘α-L-fuc residues in the paraxial mesenchyme (35). In contrast with our results, the expression of these moieties was reported by Götz and Quondumatteo (13) in human embryos.

Another tested lectin was WGA. The reaction of this lectin was general and initiated on weakly on GD10 and then continued to GD15. Since this lectin binds GlcNAc and sialic acid terminal sugars, few glycocojugates with GlcNAc or sialic acid are present in this region. The findings of Zschäbitz et al. for this sugar was negative (32), while Götz et al. have noted weak staining in the axial mesenchyme (27), which was similar to our findings. This latest investigation concluded that absent or weak WGA lectin binding in the axial mesenchyme could be related to migration, because axial mesenchymal cells no longer migrate during the early development of the vertebral column (13). Sumida et al. reported that proteins binding Con A and WGA lectins promote migration of cardiac mesenchymal cells in the chicken (36).

GSA1-B4 lectin did not react with the paraxial mesenchyme, therefore no galactose terminal sugar was obtained in this region. In contrast to our results, Zschäbitz et al. reported positive findings of paraxial mesenchyme in rats (32).

During the GDs investigated, first an unsegmented paraxial mesenchyme was derived from sclrotomal cells, then segmentation occurred rapidly for the formation of vertebral bodies, arches, and intervertebral discs (37).

According to Götz et al. the axial mesenchyme of the early human embryo is rich in sulphated glycosaminoglycans and hyaloronic acid and contains glycoproteins like fibronectin and laminin, but has no keratan sulphate (38). Toole has reported that during cell aggregation in the paraxial mesenchyme, fibronectin, tenascin, and type I collagen accumulate in the ECM by a loss of hyaluronate (39). The presence and involvement of aggrecans in the process of sclerotomal condensation around the notochord to form the vertebral body has been reported by Vasan (40). The transition to developing cartilage is marked by the deposition of cartilage matrix protein, such as proteoglycan core protein, fibronectin, chonroitin sulphate biglycan, and transients from type I and type II collagen (41). Fibronectin and tenascin collaborate in fibroblasts in culture, and this may be an important feature for regulation of cell adhesion, cell migration, and morphogenesis (42).

Although there are similarities between our findings and previous studies (13, 27, 28, 32), differences also exist, which are due to the different species examined. Species differences are a well known phenomenon in lectin histochemistry, and this can explain why our results are differ from those of Götz and Quondumatteo on human embryos (13) and Zschäbitz et al. (32) on rat embryos.

## Conclusion

According to our results, we have concluded that specific glycoconjugate molecules with GalNAc terminal sugar bind to all specific sensitive tested lectins and show temporally regulated changes, probably in the presence of components of prechondrogenic cells forming the vertebrae or in the ECM. These carbohydrate residues may be involved in phenomena such as transforming somatic epithelial cells to paraxial mesenchyme, migration and aggregation of these cells around axial organs, condensation and proliferation of these cells, and especially contributing to the conversion of mesenchymal cells to chondroblasts. Those GalNAc residues which bind WFA, SBA, and VVA lectins (in addition to the mentioned processes), may be correlated with the differentiation of chondrocytes during the development of the vertebrae. Other terminal sugars such as sialic acid, GlcNAc, galactose, and fucosylated moieties, which did not show temporally regulated changes, are probably not involved in the differentiation of the paraxial mesenchyme during early morphogenesis in vertebral column development.

Further studies are needed to understand how these specific glycoconjugates and their GalNAc terminal sugers cause morphological changes during early development of the vertebrae.

As the contribution of Shh glycoprotein has been estabilished in the development of vertebrae, further study of immunohistochemicals by specific antibodies and lectin histochemistry by tested lectins are recommended.
